# The Relationship Between Glymphatic Function, White Matter Hyperintensity and Cognition: A Structural Equation Model MRI Study

**DOI:** 10.1111/cns.70478

**Published:** 2025-06-19

**Authors:** Lin Wu, Kaixiao Chen, Zhi Zhang, Han Wang, Daojun Hong, Meng Li, Fuqing Zhou

**Affiliations:** ^1^ Jiangxi Provincial Key Laboratory for Precision Pathology and Intelligent Diagnosis, Department of Radiology, The First Affiliated Hospital, Jiangxi Medical College Nanchang University Nanchang China; ^2^ Jiangxi Province Medical Imaging Research Institute Nanchang China; ^3^ Department of Imaging Medicine Guangdong Province Traditional Chinese Medical Hospital Guangzhou China; ^4^ Department of Neurology, The First Affiliated Hospital, Jiangxi Medical College Nanchang University Nanchang China; ^5^ Department of Psychiatry and Psychotherapy Jena University Hospital Jena Germany; ^6^ Center for Intervention and Research on Adaptive and Maladaptive Brain Circuits Underlying Mental Health (C‐I‐R‐C) Halle‐Jena‐Magdeburg Germany

**Keywords:** cognition, glymphatic function, structural equation modeling, white matter hyperintensity

## Abstract

**Background:**

White matter hyperintensity (WMH) is associated with glymphatic dysfunction. Few studies have focused on the causal effects of the dysfunction of the glymphatic circulation pathways (inflow and outflow pathway) on WMH and cognitive function.

**Methods:**

This study investigated the directional effects between glymphatic circulation, WMH lesions, and cognitive function in older adults based on structural equation models. The lateral ventricle choroid plexus (ChP), the coupling strength between blood oxygen signals in gray matter and cerebrospinal fluid flow, the diffusion tensor imaging analysis along the perivascular space (DTI‐ALPS) index, and meningeal lymphatic vessels (MLVs) were analyzed for quantitative analysis of the glymphatic circulation.

**Results:**

Compared to healthy controls, participants with WMH had greater ChP volume, lower DTI‐ALPS index, and reduced MLVs function, all associated with worse cognitive performance. Both ChP/LatVent (*p* = 0.009) and DTI‐ALPS (*p* < 0.001) are significant predictors of WMH volume. Deep WMH (DWMH) partially mediated the relationship between glymphatic function (ChP/LatVent, *β* = 0.108, *p* = 0.044; DTI‐ALPS, *β* = 0.122, *p* = 0.032) and cognition. Structural equation models revealed that glymphatic outflow negatively influenced WMH (*β* = −0.572, *p* < 0.001), and WMH had a significantly negative effect on cognitive function (*β* = −0.705, *p* = 0.006).

**Conclusions:**

Our results suggest that DWMH plays a mediating role in glymphatic decline and cognitive abnormalities, and that diminished glymphatic circulation affects WMH volume, leading to decreased cognitive function.

## Graphical abstract

Participants were classified into three groups: HC (Fazekas 0), mild WMH (Fazekas 1–3), and severe WMH (Fazekas 4–6). Glymphatic function was assessed by magnetic resonance imaging. Structural equation modeling (SEM) was used to explore potential causal relationships among glymphatic inflow and outflow function, WMH volume, and cognitive performance.

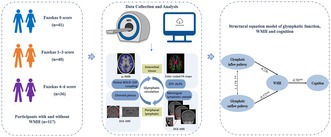

**Abbreviations**
AUC: area under the curve
BMI: body mass index
BOLD: blood oxygenation level dependent
ChP: choroid plexus
CSF: cerebrospinal fluid
DCE‐MRI: dynamic contrast‐enhanced magnetic resonance imaging
DTI‐ALPS: diffusion tensor image analysis along the perivascular space
DWMH: deep WMH
EPVS: enlarged perivascular space
FA: fractional anisotropy
HC: healthy control
ICV: intracranial volume
IQR: interquartile range
LatVent: lateral ventricle
MLVs: meningeal lymphatic vessels
MMSE: Mini‐Mental State Examination
MoCA: Montreal Cognitive Assessment
PWMH: periventricular WMH
rs‐fMRI: resting‐state functional magnetic resonance imaging
T1WI: T1‐weighted imaging
TTP: time to peak
WMH: white matter hyperintensity

## 1 Introduction

White matter hyperintensity (WMH) is defined as a point‐like or patchy hypersignal lesion in deep or periventricular white matter on T2‐weighted or fluid‐attenuated inversion recovery (FLAIR) sequences [1]. It is commonly observed in approximately 50% of individuals aged ≥ 50 years [2]. WMH is the most common imaging feature of cerebral small vessel disease; it is also linked to cognitive decline, as well as an increased risk of stroke and neuropsychological disorders, especially in aging populations [3, 4, 5]. However, due to its diverse etiology and multifactorial pathology, effective strategies for the prevention or treatment of WMH progression by managing risk factors—such as antihypertensive therapy, smoking cessation, and statin use—remain challenging [6]. As the global population ages, addressing WMH has become an urgent medical issue worldwide.

The glymphatic pathway may offer a new predictive marker and therapeutic target for managing WMH in the general aging population [7]. Our recent study, which utilized diffusion tensor image analysis along the perivascular space (DTI‐ALPS), showed that higher Fazekas scores in WMH are associated with worse glymphatic function [8]. Furthermore, a longitudinal cohort study indicated that glymphatic dysfunction could predict WMH progression [9]. The choroid plexus (ChP), a highly vascularized structure, plays a key role in cerebrospinal fluid (CSF) secretion and the filtration of substances and cytokines within the brain [10]. CSF enters the periarterial space through vasoconstriction, then travels into the interstitial brain through aquaporin 4 (AQP4) channels in glial endfoot membranes, facilitating waste excretion. Subsequently, this fluid is discharged into the perivenous space through AQP4 channels and then into the meningeal lymphatic vessels (MLVs), where it drains into extracranial deep cervical lymph nodes and participates in systemic fluid circulation [11, 12, 13, 14]. Enhanced glymphatic inflow or outflow function may have therapeutic effects in various diseases. Noninvasive 40 Hz stimulation reportedly promoted arterial‐driven CSF movement and facilitated glymphatic clearance in a mouse model of Alzheimer's disease [15, 16]. Ye et al. [17] demonstrated that borneol could improve MLVs drainage function, alleviating symptoms of Alzheimer's disease. Other areas of interest include mitigating post‐traumatic cerebral edema [18], immune inflammation [19], cerebral ischemia [20], perioperative neurocognitive disorders [21], and intracerebral hemorrhage [22]. Improving the glymphatic inflow or outflow function is expected to be a new method to control the progression of WMH. However, the relationships of WMH and dysfunction of glymphatic circulation pathways (inflow and outflow pathways) remain poorly understood.

In this study, we hypothesized that the function of the glymphatic circulation exerts a directional influence on the relationship between WMH lesion volume and cognitive function in older adults. To test this hypothesis, we investigated differences in ChP volume and permeability to indirectly characterize CSF secretion and brain lymphatic fluid flow. Neuronal activity in the brain is followed by coupled waves of blood and CSF flow [23]. The coupling between blood‐oxygenation‐level‐dependent (BOLD) signal amplitude and CSF flow was used to assess the flow dynamic function of the glymphatic circulation. For assessment along the glymphatic excretion pathway, fluid diffusion around the deep medullary vein was calculated using the DTI‐ALPS index, whereas MLVs efficiency was quantified through minimally invasive dynamic contrast‐enhanced MRI methods (Figure S1) We aimed to identify a glymphatic pathways that could slow WMH progression and cognitive decline.

## 2 Materials and Methods

### 2.1 Cohort

This study, which included intravenous injection of gadolinium contrast agent, was approved by the Ethics Committee of the First Affiliated Hospital of Nanchang University (approval number 2023‐139). All clinical investigations were conducted in accordance with the principles of the Declaration of Helsinki. Informed consent was obtained from all participants.

### 2.2 Study Participants

**FIGURE 1:**
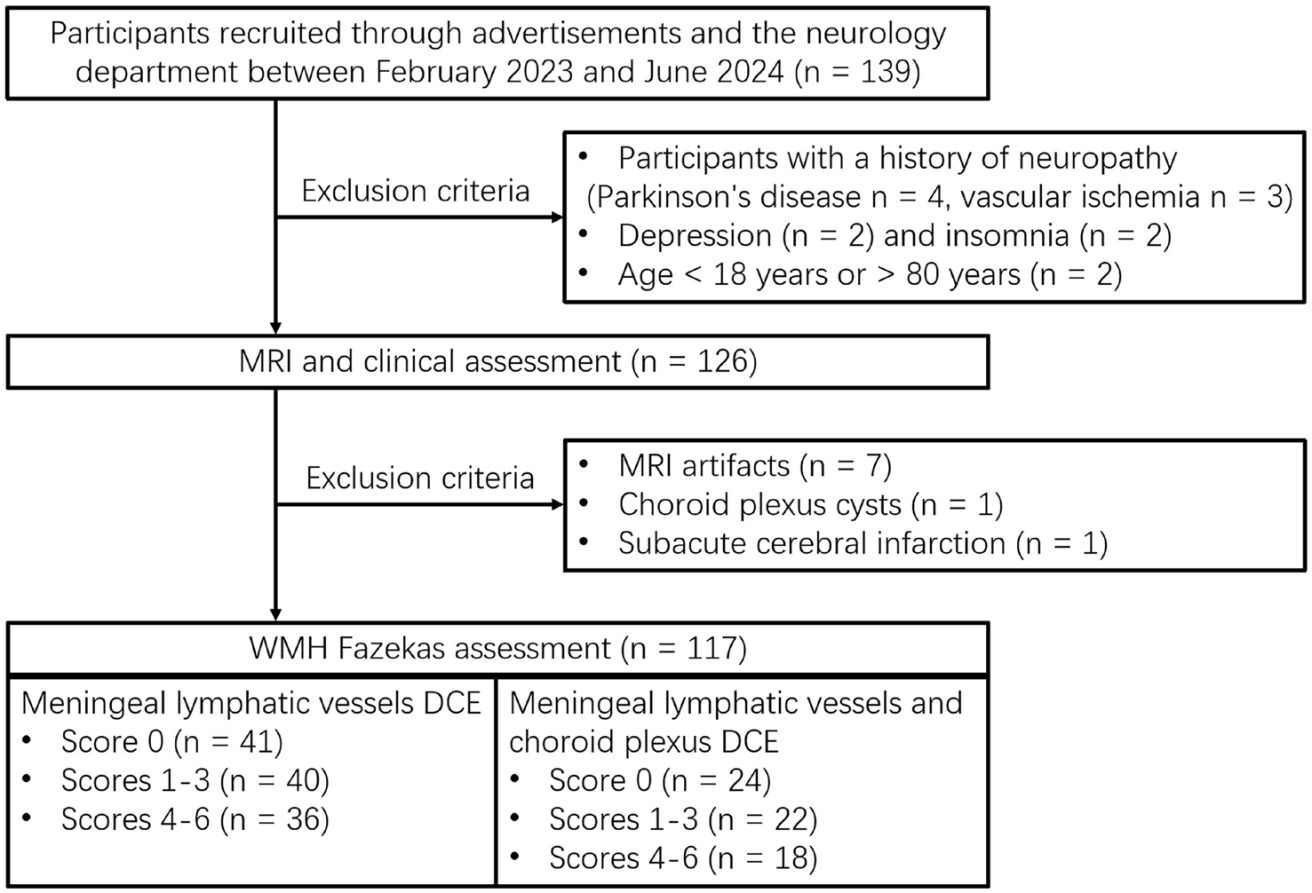
Participant flow diagram.

Patients with no significant secondary white matter lesions (such as immune, metabolic, or traumatic lesions) and no history of gadolinium allergy were enrolled. Initially, 139 participants were recruited. Exclusion criteria included: (1) dementia from causes other than Alzheimer's disease, (2) age < 18 years or > 80 years, (3) mental illness or insomnia, (4) ChP lesions or acute infarction within the past 6 months, and (5) severe MRI artifacts. 117 participants met the criteria for the study, and the subjects without WMH were classified as healthy control (HC). Sixty‐four of the 117 had dynamic contrast‐enhanced (DCE) images of both mlv and ChP (see Figure 1) All the subjects were Han Chinese from Jiangxi, China.

### 2.3 Clinical Assessment

Upon enrollment, demographic characteristics, education level, body mass index (BMI, calculated as weight in kilograms divided by height in meters squared), comorbidities, and vascular risk factors were recorded for all participants. Cognitive performance was assessed using the Montreal Cognitive Assessment (MoCA) and the Chinese version of the Mini‐Mental State Examination (MMSE) [24], with total scores ranging from 0 to 30.

### 2.4 MR Imaging Acquisition

MRI data were acquired using a Trio 3.0T MRI scanner with a 20‐channel head–neck gradient coil (Skyra, Siemens, Erlangen, Germany). For each participant, DCE imaging, high‐resolution 3D T1‐weighted imaging, diffusion tensor imaging (DTI), transverse T2‐weighted imaging, coronal T2‐FLAIR, susceptibility‐weighted imaging, and BOLD signals were obtained. The DCE protocol utilized a standard two‐dimensional T1 black‐blood sequence to acquire 23 image series for each participant; each series lasted 16.78 s. Each series included three contiguous slices with 3‐mm thickness. The contrast agent was injected at the end of the second sequence. The recommended dose (0.1 mmol/kg) of gadobutrol (Gadovist, Bayer Pharma AG) was intravenously administered using an automatic high‐pressure syringe (Spectris MRI Injector System, Medrad). Detailed MRI protocols are provided in Text S1.

### 2.5 WMH Assessment

WMH was defined as subcortical hyperintensity without cavitation on T2‐FLAIR imaging. It is further categorized as periventricular (PWMH) or deep (DWMH) according to the Fazekas scale. PWMH was graded as absent (score 0), cap (score 1), smooth halo (score 2), or irregular extension into subcortical white matter (score 3). DWMH was graded as absent (score 0), punctate (score 1), early confluent (score 2), or confluent (score 3) [25]. The PWMH and DWMH scores were then combined. Participants were classified into three groups: HC (Fazekas 0), mild WMH (Fazekas 1–3), and severe WMH (Fazekas 4–6). WMH scores were assessed by Dr. Lin Wu (MD, with 10 years of experience).

We developed a semiautomatic pipeline for WMH segmentation using nnUNet [26], which was applied to both T1‐weighted and FLAIR images; FLAIR images were resampled into T1 space. The initial model was trained on a dataset of 100 manually labeled samples. Each segmentation result was carefully inspected and corrected for inaccuracies, with a radiologist (Lin Wu) verifying accuracy. The model was retrained iteratively until visually confirmed accurate segmentation was achieved across all participants. When satisfactory performance was achieved, the model was applied to the entire cohort to generate final WMH masks. Next, morphological parameters, including center‐of‐mass coordinates, Euclidean distance to the ventricular system, and principal axis dimensions, were computed for each WMH. Lesions within 8 mm of the ventricular system were classified as periventricular, while those located more than 8 mm away were classified as deep lesions [27]. The volumes of periventricular and deep lesions were then calculated for each participant using fslstats from the FSL software suite, ensuring consistent quantification across the dataset.

### 2.6 Semimanual Segmentation Workflow and Volume Extraction for the ChP

A semimanual segmentation method, based on the protocol by Bannai et al., was used to segment the ChP. Specifically, an expert rater (Yuan Cao) who contributed to the protocol manually segmented the ChP in the lateral, third, and fourth ventricles across a dataset of 126 T1‐weighted structural images; this served as the gold standard [28]. This gold‐standard dataset was then used to train and validate an automatic segmentation model [26] via the 3D full configuration of nnUNet, with standard preprocessing steps including resampling, normalization, and data augmentation. Using the nnUNet model trained on manually segmented data, we achieved highly accurate ChP segmentation. After segmentation, we utilized fslstats from the FSL toolbox to calculate the volume of the segmented ChP in each hemisphere. To ensure precise localization and exclude any voxels from surrounding areas, inflated ventral masks generated by FreeSurfer (version 7.3.1) were applied. These segmentations were overlaid on the original T1‐weighted images and reviewed by a radiologist (Lin Wu), who manually corrected any discrepancies to maintain anatomical accuracy and consistency in segmentation boundaries. The accuracy of the deep learning method for choroid plexus segmentation was verified by comparing the results with the manual segmentation performed by the radiologist. Intermethod reliability of ChP segmentation was assessed with the use of the intra‐class correlation coefficient (ICC) (two‐way mixed model, single measure, absolute agreement). The ICC of ChP volume was 0.847 (*p* < 0.001).

### 2.7 Global BOLD‐CSF

The preprocessed resting‐state functional MRI data were analyzed using the MATLAB 2018 platform (MathWorks Inc., Natick, MA, USA) with Statistical Parametric Mapping 12. The standard preprocessing steps have been described in previous reports [29]. The main preprocessing steps included discarding the first 10 volumes of each session, slice timing correction, head motion realignment, spatial normalization, spatial smoothing with a 6‐mm kernel, and temporal filtering. The cortical gray matter was defined based on the Harvard–Oxford cortical structural atlas. BOLD and T1‐weighted imaging were used to manually define the CSF region of interest (ROI) at the bottom slices of the cerebellum. After global BOLD signals had been extracted from the cortical gray matter and CSF, the signal values were normalized by the temporal mean to reflect percentage change. To quantify coupling strength, we calculated the cross‐correlation function between global BOLD and CSF signals using Pearson's correlation; we recorded the strongest coupling coefficient for each participant. In this study, the cross‐correlation function between the negative first‐order derivative of the global BOLD signal and the CSF signal displayed a large positive peak at the zero‐time lag, and the global BOLD–CSF correlation showed a positive peak at a time lag of −6 s and a negative peak at a time lag of +2 s. We extracted the global BOLD–CSF coupling at this lag time for each participant to quantify coupling strength. We calculated these coupling intensity measures to reflect pathologically related changes in cerebrospinal fluid flow in WMH.

### 2.8 DTI‐ALPS Processing

DTI data were processed using DSI Studio software (version 2021), consistent with the methods used in our previous studies [30]. The process involved the following steps: (1) applying a mask to filter out background regions, enhance reconstruction efficiency, and conduct quality checks; (2) reconstructing diffusion data with the DTI method to characterize fiber diffusion direction; and (3) registering diffusion images to susceptibility‐weighted imaging to accurately identify brain regions containing veins and perivascular spaces. On color‐coded plots, we placed nine‐voxel (approximately 5.4 mm per side) square ROI in the regions of projection and association fibers in both cerebral hemispheres. Diffusivity values were recorded along the *x*‐axis (Dx), *y*‐axis (Dy), and *z*‐axis (Dz) within the ROI for projection and association fibers, denoted as Dxproj, Dyproj, Dxassoc, and Dzassoc, respectively. The DTI‐ALPS index was calculated as follows: DTI‐ALPS = (Dxproj + Dxassoc)/(Dyproj + Dzassoc). A low DTI‐ALPS index indicates impaired glymphatic circulation [31].

### 2.9 DCE Processing

MLVs are located around the superior sagittal sinus in the dorsal region of the brain and around the sigmoid sinus in the basal region, referred to here as dorsal MLVs and basal MLVs, respectively. To evaluate the flow functions of these regions, DCE imaging of both dorsal MLVs and basal MLVs (including left and right basal MLVs) was performed. The DCE data were analyzed using postprocessing software (syngoMMWP VE40A, Siemens AG). ROI was manually delineated by authors Kaixiao Chen and Zhi Zhang. The ROI was drawn on the basis of Ding et al. [32]. The ROI was placed on high‐signal images of the MLVs, remaining within the bright signal area and ensuring the largest possible diameter (see Figures S1 and S2). The signal strength of different voxels within ROI is averaged to generate a time‐intensity curve, which can also be called arterial input function. In our study, the arterial input function can be roughly understood as a dynamic change in the signal strength of the contrast agent inflow within MLVs. To ensure image quality, we studied patients with fitting deviations less than 0.05. The DCE data were interpreted using semiquantitative parameters derived from the time–intensity curve, including the wash‐in rate, time to peak (TTP), and area under the curve (AUC). Quantitative parameters were estimated using the extended Tofts model, which includes the volume transfer constant (*K* ^trans^), reflux rate (*k*_ep_), and volume of extravascular extracellular space (*V*_e_). *K* ^trans^ represents the diffusion rate of the gadolinium contrast agent from the vessel to surrounding tissue; reflecting contrast agent extravasation in MLVs: *V*_e_ is the volume of gadolinium contrast relative to the total extravascular extracellular space; and *k*_ep_ is *K* ^trans^/*V*_e_ [33, 34, 35] (Figure S2). Using delineated masks for the left and right ChP, mean values within these regions were recorded as the functional permeability parameters of the ChP.

### 2.10 Statistical Analysis

Demographic variables are presented as means with standard deviations or medians with interquartile ranges. Continuous and categorical variables were compared between groups using one‐way analysis of variance and the *χ* ^2^ test, respectively. Tukey post hoc analyses were used to identify the specific groups contributing to differences in clinical data, WMH and ChP volumes, global BOLD–CSF, DTI‐ALPS indices, and DCE parameters. Cohen's *d* was reported as a measure of effect size for *t*‐tests. Comparison of lymphatic circulation parameters using Analysis of Covariance (ANCOVA) and rank ANCOVA, age, sex, and education were included as covariates; Bonferroni correction was applied for multiple comparisons. Pearson correlation, Spearman correlation analysis, and robust regression analysis (RANdom SAmple Consensus) were used to assess relationships among WMH volume, ChP volume, DCE parameters, and DTI‐ALPS indices. Interrater reliability for manually defined ROIs was established using ICC. Mediation analysis and structural equation modeling (SEM) were conducted to explore mediating and directional influence relationships among glymphatic circulation, WMH, and cognitive function. The threshold for statistical significance was set at *p* < 0.05. All statistical analyses were performed by Kaixiao Chen in SPSS (version 22.0; IBM), and graphs were generated using GraphPad Prism 9.0 (GraphPad Software, San Diego, CA, USA).

### 2.11 Role of the Funding Source

The funder of the study had no role in study design, data collection, data analysis, data interpretation, or writing of the report. The corresponding authors had full access to all the data in the study and had final responsibility for the decision to submit for publication.

## 3 Results

### 3.1 Clinical and Demographic Characteristics

Age and education levels differed significantly among the groups (all *p* < 0.001). Participants in the severe WMH group were older and had lower education levels than those in the HC and mild WMH groups. There were no significant differences in sex (*p* = 0.060), BMI (*p* = 0.922), or smoking status (*p* = 0.937). The prevalences of hypertension, enlarged perivascular space (EPVS), lacunes, and microbleeds significantly varied across groups (all *p* < 0.001). MRI evaluations of EPVS, lacunes, and microbleeds are detailed in Text S2. WMH measures (PWMH volume, DWMH volume, and total WMH volume/intracranial volume [ICV]) significantly differed between the mild and severe WMH groups (*p* < 0.001). Compared with the mild WMH group, the severe WMH group had higher PWMH and DWMH volumes and greater total WMH volume/ICV. Additionally, MMSE and MoCA scores differed among the groups (all *p* < 0.001); the severe WMH group exhibited lower scores compared with the mild WMH and HC groups (Table 1).

**TABLE 1:** Participants' demographic, clinical, and cognitive characteristic.

Variable	HC (*n* = 41)	Mild WMH (*n* = 40)	Severe WMH (*n* = 36)	*p* ^a^
Age (years)	37.7 ± 8.8	46.1 ± 12.9^b^	60.1 ± 13.2^b^, ^c^	**< 0.001**
Male, *N* (%)	14 (34.1)	18 (45.0)	22 (61.1)	0.060
Education (years)	10.2 ± 4.0	9.0 ± 3.5	7.3 ± 2.6^b^, ^c^	**< 0.001**
BMI median (IQR) (kg/m^2^)	22.76 ± 1.24	22.30 ± 3.37	23.29 ± 2.40	0.922
Hypertension, *N* (%)	0 (0.0)	8 (20.0)^b^	18 (50.0)^b^, ^c^	**< 0.001**
Smoking, *N* (%)	6 (14.6)	7 (17.5)	6 (16.7)	0.937
Presence of EPVS, *N* (%)	32 (78.0)	37 (92.5)	34 (94.4)	**0.049**
Number of EPVS	2.8 ± 2.5	4.9 ± 3.4^b^	5.5 ± 3.5^b^	**< 0.001**
Presence of lacunes, *N* (%)	3 (7.3)	4 (10.0)	31 (86.1)^b^, ^c^	**< 0.001**
Presence of microbleeds, *N* (%)	0 (0.0)	1 (2.5)	16 (44.4)^b^, ^c^	**< 0.001**
WMH volume (mm^3^)				
PWMH	0 ± 0	244.33 ± 448.31^b^	8816.69 ± 8369.56^b^, ^c^	**< 0.001**
DWMH	0 ± 0	423.43 ± 587.06^b^	10368.00 ± 10398.24^b^, ^c^	**< 0.001**
Total WMH/ICV (×10^3^)	0 ± 0	0.45 ± 0.60^b^	12.56 ± 9.89^b^, ^c^	**< 0.001**
MMSE	27.7 ± 1.2	25.9 ± 1.7^b^	24.5 ± 3.8^b^	**< 0.001**
MoCA	27.0 ± 1.6	25.5 ± 2.7^b^	19.1 ± 5.9^b^, ^c^	**< 0.001**.

*Note:* Continuous variables are reported as the mean (standard deviation); otherwise, the data is the number of patients and the percentage in parentheses. Boldface indicates statistically significant results (*p* < 0.05).

^a^ *p* values represent comparisons across the three groups.

^b^ *p* < 0.05 versus HC group.

^c^ *p* < 0.05 versus mild WMH group.

### 3.2 Glymphatic Circulation Indicators

**FIGURE 2:**
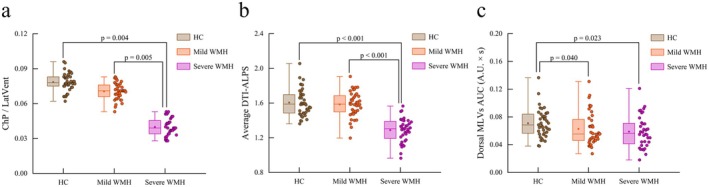
Box and whisker plots of ChP (a), dorsal MLVs (b), and DTI‐ALPS (c) data. *p* values are adjusted for age, sex, and education.

ChP measures (ChP volume, ChP/lateral ventricle [LatVent], ChP/ICV), DTI‐ALPS indices (left, right, and average of both hemispheres), and dorsal MLVs parameters (TTP, AUC, *k*_ep_) significantly differed among the groups (all *p* < 0.05) (Table 2). The reliability of manual delineation for DTI‐ALPS (*r* = 0.859, *p* < 0.001) and TTP (*r* = 0.852, *p* < 0.001) was further confirmed by ICC analysis. After adjusting for age, sex, and education, the severe WMH group showed a lower ChP/LatVent ratio compared with the mild WMH group (Cohen's *d* = 1.147, effect size *r* = 0.498) and the HC group (Cohen's *d* = 1.289, effect size *r* = 0.541) (all *p* < 0.01). Additionally, the severe WMH group had a lower average DTI‐ALPS index relative to the mild WMH group (Cohen's *d* = 2.120, effect size *r* = 0.727) and the HC group (Cohen's *d* = 2.181, effect size *r* = 0.736) (*p* < 0.001). The AUC for dorsal MLVs was greater in HC than in participants with mild WMH (Cohen's *d* = 0.391, effect size *r* = 0.192) or severe WMH (Cohen's *d* = 0.578, effect size *r* = 0.277) (all *p* < 0.05) (Figure 2, Table S1). No significant differences were found in global BOLD–CSF coupling (Figure S3).

**TABLE 2:** Comparison of glymphatic circulation indicators among the study groups.

Variable	HC	Mild WMH	Severe WMH	*p*
ChP				
ChP volume	859.29 ± 451.89	1092.43 ± 552.11	1194.06 ± 611.25	**0.022**
ChP/LatVent	0.079 ± 0.034	0.071 ± 0.028	0.040 ± 0.026	**< 0.001**
ChP/ICV (×10^−3^)	0.59 ± 0.31	0.72 ± 0.33	0.78 ± 0.38	**0.045**
Global BOLD–CSF coupling				
Maximum strength	−0.227 ± 0.137	−0.206 ± 0.141	−0.187 ± 0.092	0.363
+2 s lag strength	−0.088 ± 0.175	−0.188 ± 0.194	−0.038 ± 0.146	0.181
−6 s lag strength	0.049 ± 0.169	0.081 ± 0.127	0.020 ± 0.166	0.250
d(globalBOLD)/dt coupling strength	0.069 ± 0.185	0.108 ± 0.203	0.063 ± 0.172	0.516
DTI‐ALPS				
Left	1.606 ± 0.182	1.579 ± 0.162	1.270 ± 0.169	**< 0.001**
Right	1.606 ± 0.193	1.591 ± 0.194	1.304 ± 0.167	**< 0.001**
Bilateral average	1.606 ± 0.155	1.585 ± 0.144	1.287 ± 0.137	**< 0.001**
Dorsal MLVs				
Wash–in rate	0.086 ± 0.023	0.080 ± 0.029	0.075 ± 0.030	0.208
TTP	1.038 ± 0.224	1.128 ± 0.300	1.214 ± 0.379	**0.033**
AUC	0.072 ± 0.022	0.063 ± 0.024	0.059 ± 0.023	**0.028**
*K* ^trans^	0.053 ± 0.017	0.049 ± 0.017	0.046 ± 0.016	0.160
*k*_ep_	0.495 ± 0.183	0.449 ± 0.141	0.400 ± 0.158	**0.039**
*V*_e_	0.121 ± 0.041	0.123 ± 0.054	0.155 ± 0.150	0.203
Left basal MLVs				
Wash–in rate	0.110 ± 0.047	0.126 ± 0.101	0.098 ± 0.043	0.217
TTP	0.983 ± 0.199	0.952 ± 0.208	1.004 ± 0.356	0.677
AUC	0.090 ± 0.036	0.095 ± 0.050	0.079 ± 0.039	0.230
*K* ^trans^	0.069 ± 0.029	0.071 ± 0.035	0.060 ± 0.028	0.238
*k*_ep_	0.593 ± 0.128	0.583 ± 0.134	0.549 ± 0.285	0.075
*V*_e_	0.117 ± 0.044	0.120 ± 0.050	0.117 ± 0.051	0.960
Right basal MLVs				
Wash–in rate	0.126 ± 0.059	0.109 ± 0.054	0.106 ± 0.048	0.215
TTP	0.915 ± 0.177	0.973 ± 0.276	1.067 ± 0.500	0.203
AUC	0.106 ± 0.051	0.091 ± 0.048	0.087 ± 0.043	0.165
*K* ^trans^	0.080 ± 0.038	0.068 ± 0.035	0.066 ± 0.031	0.159
*k*_ep_	0.612 ± 0.168	0.565 ± 0.148	0.564 ± 0.199	0.377
*V*_e_	0.132 ± 0.059	0.118 ± 0.049	0.122 ± 0.051	0.496

*Note:* *p* values are for comparisons across the three groups. Boldface indicates statistically significant results (*p* < 0.05).

### 3.3 Relationships of Glymphatic Circulation Indicators With WMH and Cognition

Among participants with WMH (including mild and severe WMH, *n* = 76), ChP/LatVent was negatively correlated with total WMH volume (*r* = −0.404, *p* < 0.001), PWMH volume (*r* = −0.347, *p* = 0.002), DWMH volume (*r* = −0.430, *p* < 0.001), and total WMH volume/ICV (*r* = −0.395, *p* < 0.001). It was positively correlated with MMSE scores (*r* = 0.377, *p* < 0.001) and MoCA scores (*r* = 0.436, *p* < 0.001) (Figure S4a–f). The average DTI‐ALPS index was negatively correlated with total WMH volume, PWMH volume, DWMH volume, and total WMH volume/ICV (all *p* < 0.001). It was positively correlated with MMSE and MoCA scores (both *p* < 0.001) (Figure S4g–l). The AUC of dorsal MLVs was not significantly correlated with either WMH volume or cognitive scores.

In the robust regression analysis, age, sex, and education level were included as control variables. When the global BOLD–CSF coupling value was added to the hierarchical model containing ChP volume measurements, the results indicated that ChP/LatVent (*p* = 0.009) was a significant predictor of WMH (Table S2). In a separate regression model including dorsal MLVs parameters (wash‐in, TTP, AUC, *K* ^trans^, *k*_ep_, *V*_e_), the average DTI‐ALPS (*p* < 0.001) emerged as a significant predictor of WMH (Table S3).

### 3.4 ChP Volume and Permeability

Among the 64 participants with DCE images of both MLVs and ChP, the average *K* ^trans^ value of the bilateral ChP was correlated with ChP/LatVent (*r* = 0.257, *p* = 0.044). In participants with mild WMH (*n* = 22), ChP/LatVent was positively correlated with the average permeability indices of the bilateral ChP, including the average wash‐in coefficient (*r* = 0.505, *p* = 0.019), average AUC (*r* = 0.531, *p* = 0.013), and average *K* ^trans^ (*r* = 0.527, *p* = 0.014) (Figure S4m–o, Table S4).

### 3.5 Mediation Effect Analysis

**FIGURE 3:**
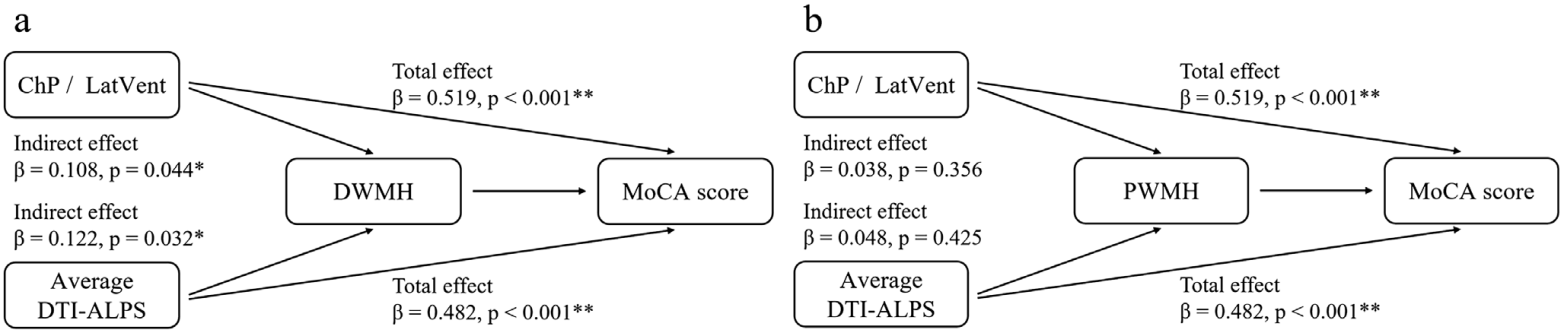
Mediation effect of WMH volume on the relationship between glymphatic inflow/outflow and cognition. (a) DWMH partially mediated the association between glymphatic circulation and MoCA scores. (b) PWMH did not significantly mediate the relationship between glymphatic function and MoCA scores. *p < 0.05; **p < 0.001.

In the mediation analysis, when the independent variables ChP/LatVent and bilateral average DTI‐ALPS were analyzed separately, DWMH emerged as a significant partial mediator between ChP/LatVent and MoCA scores (*β* = 0.108, *p* = 0.044) and between average DTI‐ALPS and MoCA scores (*β* = 0.122, *p* = 0.032) (Figure 3a). In contrast, PWMH did not significantly mediate the relationship between ChP volume and cognition or between DTI‐ALPS and cognition (Figure 3b).

### 3.6 Structural Equation Model of Glymphatic Function, WMH and Cognition

**FIGURE 4:**
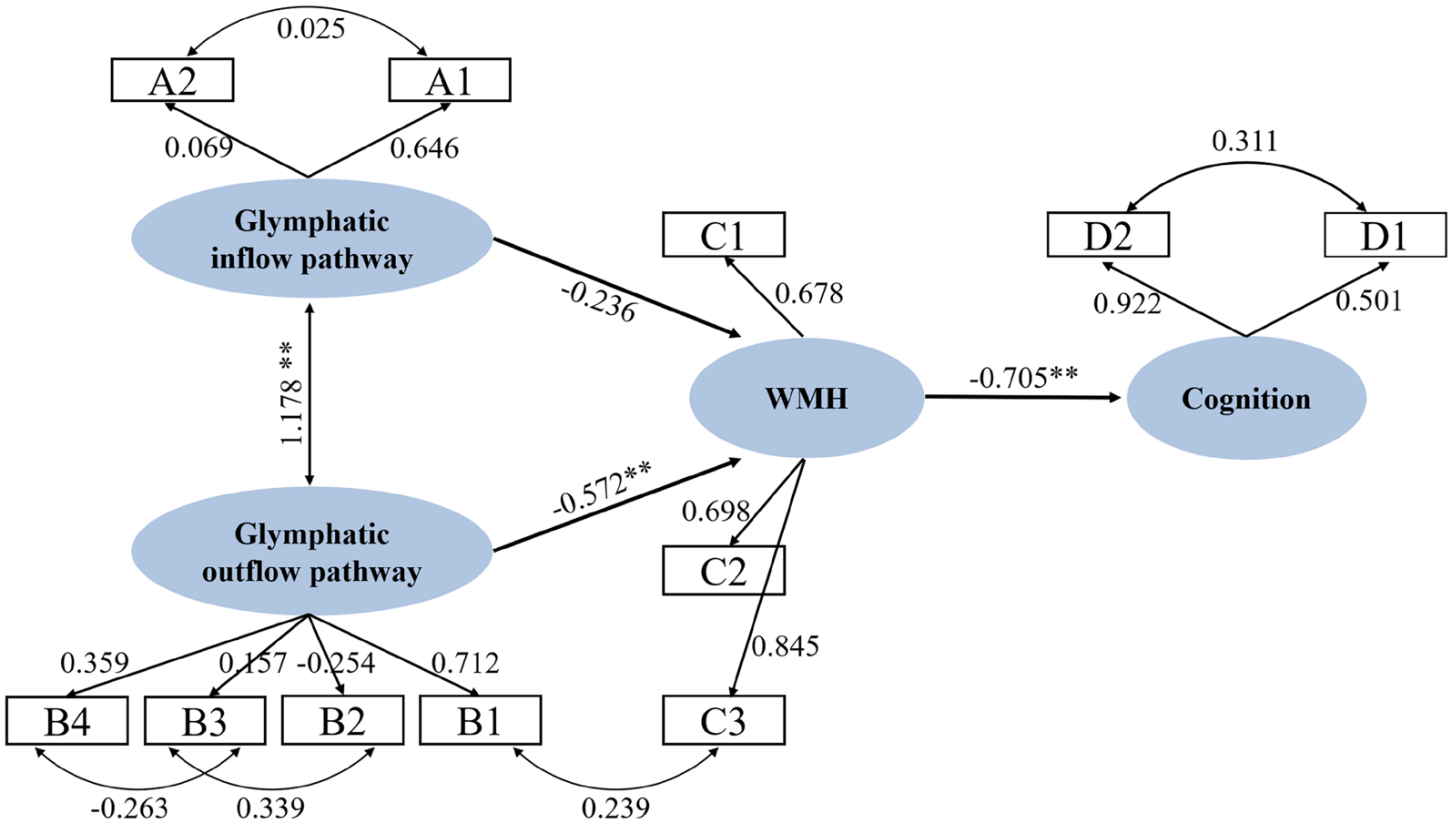
Structural equation model. A1: ChP/LatVent A2: Maximum strength of global BOLD–CSF coupling; B1: Average‐DTI‐ALPS, B2: Dorsal TTP, B3: Dorsal AUC, B4: Dorsal Kep; C1: PWMH, C2:DWMH, C3:Fazekas score; D1: MMSE, D2: MoCA. “**” indicates statistically significant results (*p* < 0.01).

In this study, we developed an SEM with a good fit (*χ* ^2^ = 44.053, df = 33, *χ* ^2^/df = 1.335, comparative fit index = 0.962, goodness of fit index = 0.914, root mean square error of approximation = 0.068). ChP/LatVent (*β* = 0.646) and bilateral average DTI‐ALPS value (*β* = 0.712) showed a higher factor load in the latent variable of the glymphatic circulation. The path coefficient indicated that the evaluation indexes based on the glymphatic outflow path had a significant negative effect on WMH (*β* = −0.572, *p* < 0.001) and WMH had a significant negative effect on cognitive function (*β* = −0.705, *p* = 0.006) (Figure 4).

## 4 Discussion

This study was conducted to enhance the understanding of WMH pathogenesis through a multivariate analysis approach, using SEM to clarify directional relationships among glymphatic circulation, WMH volume, and cognitive variables. We found that WMH is associated with abnormal glymphatic function, and that glymphatic decline is positively correlated with cognitive impairment. Compared with PWMH, DWMH serve as a mediator between glymphatic decline and cognitive abnormalities. SEM also revealed a directional relationship: decreased glymphatic outflow function negatively impacts WMH, which then negatively affects cognition. Additionally, functional changes in MLVs, which excrete metabolic waste to the extracranial deep cervical lymph nodes, are not synchronized. In the severe WMH group, dorsal MLVs function declines earlier than basal MLVs function. Analysis of the structure and function indicators of ChP across the three groups showed that an increase in ChP volume was nonlinearly correlated with a decrease in osmotic function.

We found a directional relationship among glymphatic function, WMH, and cognition. The Bootstrap method was employed to robustly assess the structural equation model. The findings also indicated that the index evaluating glymphatic outflow path had a significantly negative effect on WMH (*β* = −0.683, 95% CI [−0.977, −0.401]). The factor loading of ALPS on glymphatic efflux was greater than that of MLVs. Previous studies have revealed correlations among ALPS volume, WMH volume, and cognitive function [8, 36, 37]. Although some researchers have questioned the significance of ALPS, an increasing number of studies have shown that it constitutes an important noninvasive indicator of glymphatic function [38]. We speculate that decreased glymphatic circulation accelerates the increase in WMH volume and contributes to cognitive decline in the WMH population. Further investigation is needed to determine whether enhancement of glymphatic outflow can slow WMH volume progression. Additionally, we subdivided WMH volume in the deep and lateral ventricles based on distance from the lateral ventricle; we found that only DWMH mediated the relationship between glymphatic function and cognition. The pathophysiological mechanisms of DWMH and PWMH appear to differ. DWMH is frequently connected to EPVS; larger DWMH is associated with more connected PVS [39]. Because perivascular space is central structures in the glymphatic system [11, 40], this morphological relationship suggests that DWMH formation and expansion are closely related to glymphatic function.

Functional abnormalities in basal and dorsal MLVs differ in WMH. MLVs are a primary downstream structure of the glymphatic system, facilitating meningeal glymphatic drainage and aiding in the clearance of neurotoxic substances [41]. The functional decline of MLVs in different regions during aging is not synchronized, reflecting structural and functional differences. In mice, basal MLVs show only minor age‐related changes in density and diameter relative to dorsal MLVs [42]. This difference in degeneration is likely related to morphological distinctions between dorsal and basal MLVs [42]. Dorsal MLVs predominantly display a continuously sealed, zipper‐like junctional pattern with immature morphology, which contributes to fluid drainage and immune responses in intracranial tumors [43]. In contrast, basal MLVs exhibit a loose, button‐like endothelial cell morphology and unique anatomical location, making them a major pathway for CSF outflow and clearance [42]. Recent research has shown that disruption of dorsal MLVs alone can impair fluid drainage and may even influence downstream neuroinflammation without affecting basal MLVs [44]. This age‐related discrepancy in functional decline between dorsal and basal MLVs may result from compensatory lymphoproliferative mechanisms in basal MLVs. Such a notion aligns with our finding that dorsal MLVs function declines earlier than basal MLVs function in aging patients with WMH.

The relationship between ChP volume and permeability during aging remains unclear. Our results showed a correlation between ChP volume (as a ratio of lateral ventricle volume) and ChP permeability in mild WMH. ChP abnormalities are not solely secondary to lateral ventricle enlargement; they also involve independent processes [45]. In a study regarding the clinical spectrum of Alzheimer's disease, a correlation between ChP volume and permeability was identified only in patients with mild cognitive impairment, but not in the early (subjective cognitive impairment) or late (Alzheimer's disease) stages. This finding is consistent with our results—we found no correlation in the HC or severe WMH group. In addition to aging, various factors (e.g., hypertension, cerebral perfusion, and structural calcification) influence changes in the ChP. For example, arterial hypertension can increase ChP volume and disrupt the blood–CSF barrier in the ChP region [46]. We speculate that the relationship between age‐related declines in ChP permeability and ChP volume is more complex than a simple linear correlation. The research by Song et al. [47] revealed both depleted and enriched gut microbes in cerebral small vessel disease with WMH, and the affected metabolites were related to the patients' cognition, providing a comprehensive framework for understanding the role of the microbiota‐gut‐brain axis in the pathophysiological mechanism of cerebrovascular diseases. This factor should also be taken into account in future related studies.

Low‐frequency intrinsic vasomotor contractions can produce substantial CSF flow [23]. Compared with cardiac pulsation, these contractions have a much greater effect on CSF inflow during wakefulness and serve as another critical driving force [48]. Oscillations in the global BOLD signal at low frequencies (< 0.1 Hz) are associated with slow vasomotor pulsations, which are thought to drive CSF flow and are closely linked to glymphatic clearance. Reduced global BOLD–CSF coupling has been observed in individuals with small cerebral vessel disease [29], depressive disorder with sleep disturbance [49] and Parkinson's disease [50]; altered CSF flow is associated with disease severity. We found a gradual decline in global BOLD–CSF coupling strength from HC to participants with severe WMH, although the difference was not statistically significant. The region near the bottom of the cerebellum is expected to have the highest sensitivity to inflow effects. We selected ROI from three different slices near the bottom layer of the cerebellum, and the statistical results were consistent (see Figure S3). In our study, the acquisition of a brain volume fMRI signal required 2 s, which may have resulted in the aliasing of faster pulsations into slower frequencies. The ultrafast magnetic resonance encephalography technique, which captures whole‐brain images in 100 ms, can eliminate aliasing effects and may serve as a novel method to comprehensively monitor physiological pulsations within brain tissue in future WMH studies.

Our study had several limitations. First, while noninvasive MRI assessment of glymphatic inflow is advantageous in vivo, it presents challenges. Three physiological mechanisms—cardiac, respiratory, and very low‐frequency pulsations—affect CSF propulsion. These types of pulsations have distinct spatiotemporal patterns and coexist [51]. Future research should consider the effects of different physiological variations in CSF pulsation on glymphatic circulation. Second, hypertension is associated with increased WMH volume. In our study, we observed statistically significant differences in hypertension prevalence among HC, individuals with mild WMH, and individuals with severe WMH. We used two‐way analysis of variance to examine the effect of hypertension on glymphatic circulation. The main effect analysis suggested that hypertension had no impact on ChP volume (*F* = 1.190, *p* = 0.703) or dorsal MLVs (AUC, *F* = 0.028, *p* = 0.703). Further investigation is warranted to determine whether hypertension affects WMH volume through altered glymphatic function. Third, ChP volume/permeability, global BOLD‐CSF, and ALPS index are indirect measures for assessing glymphatic circulation. In the structural equation model analysis of this study, we divided these indicators into the function of glymphatic inflow and glymphatic outflow, which has certain limitations. However, this classification is defined in terms of the circulatory direction of the glymphatic pathway and can also be used to compare the robustness of the results of similar studies. In the future, other more recognized technologies that directly respond to glymphatic function (such as intrathecal injection of gadolinium) should be considered. Fourth, severe WMH may also be the prodromal stage of degenerative diseases. With age and neurodegeneration, the radial asymmetry within the white matter tract decreases, mirroring the effects that would occur to ALPS index due to alterations in the diffusivity within perivascular space [52]. This study does not rule out that abnormal ALPS index in severe WMH is driven by degenerative diseases.

## 5 Conclusion

Glymphatic dysfunction is associated with WMH severity and cognitive decline. DWMH mediates the relationship between glymphatic function and cognitive abnormalities. A directional relationship was observed: decreased glymphatic circulation affects WMH volume, and WMH negatively impacts cognitive function. Further validation and longitudinal studies are needed to determine whether the enhancement of glymphatic efflux can control WMH progression. Our finding can provide a basis for future clinicians to improve glymphatic function as a potential target for prevention and control of WMH.

## Author Contributions

L.W. and F.Z. designed the study. K.C. and Z.Z. recruited subjects, conducted the experiments, performed the statistical analysis, and drafted the manuscript. H.W. and D.H. participated in the recruitment of subjects and contributed to clinical data acquisition. M.L. contributed to the analysis of the structural MRI data. L.W., M.L., and F.Z. revised the manuscript. All authors contributed to the article and approved the submitted version.

## Disclosure

Associated data: This section collects any data citations, data availability statements, or Supporting Information included in this article.

## Ethics Statement

This study was approved by the Medical Ethics Committee of the First Affiliated Hospital of Nanchang University.

## Consent

All participants gave their written informed consent.

## Conflicts of Interest

The authors declare no conflicts of interest.

## Supporting information


**Figure S1.** Schematic of the study workflow showing methods for assessment of glymphatic circulation.
**Figure S2.** Semiquantitative and fully quantitative evaluation of meningeal lymphatic vessel function via DCE‐MRI.
**Figure S3.** Three sections of CSF signal extraction from the cerebellar substratum and box plot of the global BOLD–CSF coupling coefficient.
**Figure S4.** Relationships of glymphatic circulation indicators with WMH and cognition.
**Table S1.** Comparison of glymphatic circulation indicators among study groups.
**Table S2.** Multivariate linear regression analysis of glymphatic inflow in the WMH group.
**Table S3.** Multivariate linear regression analysis of glymphatic outflow in the WMH group.
**Table S4.** Relationships between ChP/LatVent value and ChP permeability in each group.
**Text S1.** MRI protocol used in our study.
**Text S2.** MRI evaluation of enlarged perivascular space, lacunes, and microbleeds.

## Data Availability

The data collected in this study, including de‐identified participant data and the data dictionary are available to researchers through corresponding author Prof. Fuqing Zhou upon reasonable request.

## References

[1] J. M. Wardlaw , E. E. Smith , G. J. Biessels , et al., “Neuroimaging Standards for Research Into Small Vessel Disease and Its Contribution to Ageing and Neurodegeneration,” Lancet Neurology 12, no. 8 (2013): 822–838, 10.1016/S1474-4422(13)70124-8. 23867200 PMC3714437

[2] E. Garde , E. L. Mortensen , K. Krabbe , E. Rostrup , and H. B. W. Larsson , “Relation Between Age‐Related Decline in Intelligence and Cerebral White‐Matter Hyperintensities in Healthy Octogenarians: A Longitudinal Study,” Lancet 356, no. 9230 (2000): 628–634, 10.1016/S0140-6736(00)02604-0. 10968435

[3] N. D. Prins and P. Scheltens , “White Matter Hyperintensities, Cognitive Impairment and Dementia: An Update,” Nature Reviews. Neurology 11, no. 3 (2015): 157–165, 10.1038/nrneurol.2015.10. 25686760

[4] A. Amin Al Olama , J. M. S. Wason , A. M. Tuladhar , et al., “Simple MRI Score Aids Prediction of Dementia in Cerebral Small Vessel Disease,” Neurology 94, no. 12 (2020): e1294–e1302, 10.1212/WNL.0000000000009141. 32123050 PMC7274929

[5] J. Alber , S. Alladi , H. J. Bae , et al., “White Matter Hyperintensities in Vascular Contributions to Cognitive Impairment and Dementia (VCID): Knowledge Gaps and Opportunities,” Alzheimers Dement 5 (2019): 107–117, 10.1016/j.trci.2019.02.001. PMC6461571 31011621

[6] T. P. Ottavi , E. Pepper , G. Bateman , M. Fiorentino , and A. Brodtmann , “Consensus Statement for the Management of Incidentally Found Brain White Matter Hyperintensities in General Medical Practice,” Medical Journal of Australia 219, no. 6 (2023): 278–284, 10.5694/mja2.52079. 37604652

[7] Y. Tian , X. Cai , Y. Zhou , et al., “Impaired Glymphatic System as Evidenced by Low Diffusivity Along Perivascular Spaces Is Associated With Cerebral Small Vessel Disease: A Population‐Based Study,” Stroke and Vascular Neurology 8, no. 5 (2023): 413–423, 10.1136/svn-2022-002191. 37045543 PMC10647865

[8] Z. Zhi , X. Liang , M. Huang , L. Wu , and F. Zhou , “The Association Between Glymphatic System Dysfunction and Alterations in Cerebral Function and Structure in Patients With White Matter Hyperintensities,” Neuroreport 35, no. 7 (2024): 476–485, 10.1097/WNR.0000000000002031. 38597326

[9] Y. Zhou , R. Xue , Y. Li , et al., “Impaired Meningeal Lymphatics and Glymphatic Pathway in Patients With White Matter Hyperintensity,” Advanced Science 11, no. 26 (2024): e2402059, 10.1002/advs.202402059. 38704728 PMC11234435

[10] M. P. Lun , E. S. Monuki , M. K. Lehtinen , et al., “Development and Functions of the Choroid Plexus‐Cerebrospinal Fluid System,” Nature Reviews Neuroscience 16, no. 8 (2015): 445–457, 10.1038/nrn3921. 26174708 PMC4629451

[11] J. J. Iliff , M. Wang , Y. Liao , et al., “A Paravascular Pathway Facilitates CSF Flow Through the Brain Parenchyma and the Clearance of Interstitial Solutes, Including Amyloid β,” Science Translational Medicine 4, no. 147 (2012): 147ra111, 10.1126/scitranslmed.3003748. PMC3551275 22896675

[12] S. T. Proulx , “Cerebrospinal Fluid Outflow: A Review of the Historical and Contemporary Evidence for Arachnoid Villi, Perineural Routes, and Dural Lymphatics,” Cellular and Molecular Life Sciences 78, no. 6 (2021): 2429–2457, 10.1007/s00018-020-03706-5. 33427948 PMC8004496

[13] J. H. Yoon , H. Jin , H. J. Kim , et al., “Nasopharyngeal Lymphatic Plexus Is a Hub for Cerebrospinal Fluid Drainage,” Nature 625, no. 7996 (2024): 768–777, 10.1038/s41586-023-06899-4. 38200313 PMC10808075

[14] T. Bohr , P. G. Hjorth , S. C. Holst , et al., “The Glymphatic System: Current Understanding and Modeling,” IScience 25, no. 9 (2022): 104987, 10.1016/j.isci.2022.104987. 36093063 PMC9460186

[15] M. H. Murdock , C. Y. Yang , N. Sun , et al., “Multisensory Gamma Stimulation Promotes Glymphatic Clearance of Amyloid,” Nature 627, no. 8002 (2024): 149–156, 10.1038/s41586-024-07132-6. 38418876 PMC10917684

[16] L. F. Jiang‐Xie , A. Drieu , K. Bhasiin , D. Quintero , I. Smirnov , and J. Kipnis , “Neuronal Dynamics Direct Cerebrospinal Fluid Perfusion and Brain Clearance,” Nature 627, no. 8002 (2024): 157–164, 10.1038/s41586-024-07108-6. 38418877 PMC12054998

[17] T. Ye , X. Yan , H. Bai , et al., “Borneol Regulates Meningeal Lymphatic Valve Plasticity to Clear Aβ Aggregates in the Prevention of AD‐Like Symptoms,” Phytomedicine 130 (2024): 155753, 10.1016/j.phymed.2024.155753. 38795693

[18] R. Hussain , J. Tithof , W. Wang , et al., “Potentiating Glymphatic Drainage Minimizes Post‐Traumatic Cerebral Oedema,” Nature 623, no. 7989 (2023): 992–1000, 10.1038/s41586-023-06737-7. 37968397 PMC11216305

[19] W. Li , B. Sun , X. Zhang , et al., “Near‐Infrared‐II Imaging Revealed Hypothermia Regulates Neuroinflammation Following Brain Injury by Increasing the Glymphatic Influx,” ACS Nano 18, no. 21 (2024): 13836–13848, 10.1021/acsnano.4c02652. 38753820

[20] B. Sun , D. Fang , W. Li , M. Li , and S. Zhu , “NIR‐II Nanoprobes for Investigating the Glymphatic System Function Under Anesthesia and Stroke Injury,” Journal of Nanobiotechnology 22, no. 1 (2024): 200, 10.1186/s12951-024-02481-w. 38654299 PMC11040925

[21] R. Dong , Y. Han , P. Lv , et al., “Long‐Term Isoflurane Anesthesia Induces Cognitive Deficits via AQP4 Depolarization Mediated Blunted Glymphatic Inflammatory Proteins Clearance,” Journal of Cerebral Blood Flow and Metabolism 44, no. 8 (2024): 1450–1466, 10.1177/0271678X241237073. 38443763 PMC11342724

[22] H. H. Tsai , Y. C. Hsieh , J. S. Lin , et al., “Functional Investigation of Meningeal Lymphatic System in Experimental Intracerebral Hemorrhage,” Stroke 53, no. 3 (2022): 987–998, 10.1161/STROKEAHA.121.037834. 35144488

[23] N. E. Fultz , G. Bonmassar , K. Setsompop , et al., “Coupled Electrophysiological, Hemodynamic, and Cerebrospinal Fluid Oscillations in Human Sleep,” Science 366, no. 6465 (2019): 628–631, 10.1126/science.aax5440. 31672896 PMC7309589

[24] M. Y. Zhang , R. Katzman , D. Salmon , et al., “The Prevalence of Dementia and Alzheimer's Disease in Shanghai, China: Impact of Age, Gender, and Education,” Annals of Neurology 27, no. 4 (1990): 428–437, 10.1002/ana.410270412. 2353798

[25] F. Fazekas , K. Niederkorn , R. Schmidt , et al., “White Matter Signal Abnormalities in Normal Individuals: Correlation With Carotid Ultrasonography, Cerebral Blood Flow Measurements, and Cerebrovascular Risk Factors,” Stroke 19, no. 10 (1988): 1285–1288, 10.1161/01.str.19.10.1285. 3051534

[26] F. Isensee , P. F. Jaeger , S. A. A. Kohl , J. Petersen , and K. H. Maier‐Hein , “nnU‐Net: A Self‐Configuring Method for Deep Learning‐Based Biomedical Image Segmentation,” Nature Methods 18, no. 2 (2021): 203–211, 10.1038/s41592-020-01008-z. 33288961

[27] A. M. Brickman , J. R. Sneed , F. A. Provenzano , et al., “Quantitative Approaches for Assessment of White Matter Hyperintensities in Elderly Populations,” Psychiatry Research 193, no. 2 (2011): 101–106, 10.1016/j.pscychresns.2011.03.007. 21680159 PMC3164869

[28] D. Bannai , Y. Cao , M. Keshavan , M. Reuter , and P. Lizano , “Manual Segmentation of the Human Choroid Plexus Using Brain MRI,” Journal of Visualized Experiments 202 (2023): e65341, 10.3791/65341. PMC13334355 38163279

[29] Y. Zhang , R. Zhang , S. Wang , et al., “Reduced Coupling Between the Global Blood‐Oxygen‐Level‐Dependent Signal and Cerebrospinal Fluid Inflow Is Associated With the Severity of Small Vessel Disease,” NeuroImage: Clinical 36 (2022): 103229, 10.1016/j.nicl.2022.103229. 36252555 PMC9668594

[30] L. Wu , Z. Zhang , X. Liang , et al., “Glymphatic System Dysfunction in Recovered Patients With Mild COVID‐19: A DTI‐ALPS Study,” iScience 27, no. 1 (2024): 108647, 10.1016/j.isci.2023.108647. 38155770 PMC10753064

[31] T. Taoka , Y. Masutani , H. Kawai , et al., “Evaluation of Glymphatic System Activity With the Diffusion MR Technique: Diffusion Tensor Image Analysis Along the Perivascular Space (DTI‐ALPS) in Alzheimer's Disease Cases,” Japanese Journal of Radiology 35, no. 4 (2017): 172–178, 10.1007/s11604-017-0617-z. 28197821

[32] X. B. Ding , X. X. Wang , D. H. Xia , et al., “Impaired Meningeal Lymphatic Drainage in Patients With Idiopathic Parkinson's Disease,” Nature Medicine 27, no. 3 (2021): 411–418, 10.1038/s41591-020-01198-1. 33462448

[33] M. Bergamino , L. Bonzano , F. Levrero , G. L. Mancardi , and L. Roccatagliata , “A Review of Technical Aspects of T1‐Weighted Dynamic Contrast‐Enhanced Magnetic Resonance Imaging (DCE‐MRI) in Human Brain Tumors,” Physica Medica 30, no. 6 (2014): 635–643, 10.1016/j.ejmp.2014.04.005. 24793824

[34] Z. Kong , C. Yan , R. Zhu , et al., “Imaging Biomarkers Guided Anti‐Angiogenic Therapy for Malignant Gliomas,” NeuroImage: Clinical 20 (2018): 51–60, 10.1016/j.nicl.2018.07.001. 30069427 PMC6067083

[35] L. Hu , Y. F. Zha , L. Wang , et al., “Quantitative Evaluation of Vertebral Microvascular Permeability and Fat Fraction in Alloxan‐Induced Diabetic Rabbits,” Radiology 287, no. 1 (2018): 128–136, 10.1148/radiol.2017170760. 29156149

[36] H. Li , M. A. Jacob , M. Cai , et al., “Perivascular Spaces, Diffusivity Along Perivascular Spaces, and Free Water in Cerebral Small Vessel Disease,” Neurology 102, no. 9 (2024): e209306, 10.1212/WNL.0000000000209306. 38626373

[37] W. Zeng , Y. Chen , Z. Zhu , et al., “Severity of White Matter Hyperintensities: Lesion Patterns, Cognition, and Microstructural Changes,” Journal of Cerebral Blood Flow and Metabolism 40, no. 12 (2020): 2454–2463, 10.1177/0271678X19893600. 31865841 PMC7820685

[38] T. Taoka , R. Ito , R. Nakamichi , T. Nakane , H. Kawai , and S. Naganawa , “Diffusion Tensor Image Analysis ALong the Perivascular Space (DTI‐ALPS): Revisiting the Meaning and Significance of the Method,” Magnetic Resonance in Medical Sciences 23, no. 3 (2024): 268–290, 10.2463/mrms.rev.2023-0175. 38569866 PMC11234944

[39] Y. Huo , Y. Wang , C. Guo , et al., “Deep White Matter Hyperintensity Is Spatially Correlated to MRI‐Visible Perivascular Spaces in Cerebral Small Vessel Disease on 7 Tesla MRI,” Stroke and Vascular Neurology 8, no. 2 (2023): 144–150, 10.1136/svn-2022-001611. 36170993 PMC10176991

[40] J. M. Wardlaw , H. Benveniste , M. Nedergaard , et al., “Perivascular Spaces in the Brain: Anatomy, Physiology and Pathology,” Nature Reviews. Neurology 16, no. 3 (2020): 137–153, 10.1038/s41582-020-0312-z. 32094487

[41] S. Da Mesquita , A. Louveau , A. Vaccari , et al., “Functional Aspects of Meningeal Lymphatics in Ageing and Alzheimer's Disease,” Nature 560, no. 7717 (2018): 185–191, 10.1038/s41586-018-0368-8. 30046111 PMC6085146

[42] J. H. Ahn , H. Cho , J. H. Kim , et al., “Meningeal Lymphatic Vessels at the Skull Base Drain Cerebrospinal Fluid,” Nature 572, no. 7767 (2019): 62–66, 10.1038/s41586-019-1419-5. 31341278

[43] X. Hu , Q. Deng , L. Ma , et al., “Meningeal Lymphatic Vessels Regulate Brain Tumor Drainage and Immunity,” Cell Research 30, no. 3 (2020): 229–243, 10.1038/s41422-020-0287-8. 32094452 PMC7054407

[44] C. H. Wu , F. C. Chang , Y. F. Wang , et al., “Impaired Glymphatic and Meningeal Lymphatic Functions in Patients With Chronic Migraine,” Annals of Neurology 95, no. 3 (2024): 583–595, 10.1002/ana.26842. 38055324

[45] J. D. Choi , Y. Moon , H. J. Kim , Y. Yim , S. Lee , and W. J. Moon , “Choroid Plexus Volume and Permeability at Brain MRI Within the Alzheimer Disease Clinical Spectrum,” Radiology 304, no. 3 (2022): 635–645, 10.1148/radiol.212400. 35579521

[46] S. W. Bothwell , D. Janigro , and A. Patabendige , “Cerebrospinal Fluid Dynamics and Intracranial Pressure Elevation in Neurological Diseases,” Fluids and Barriers of the CNS 16, no. 1 (2019): 9, 10.1186/s12987-019-0129-6. 30967147 PMC6456952

[47] Y. Song , X. Zhou , H. Zhao , et al., “Characterizing the Role of the Microbiota‐Gut‐Brain Axis in Cerebral Small Vessel Disease: An Integrative Multi‐Omics Study,” NeuroImage 303, no. 1 (2024): 120918, 10.1016/j.neuroimage.2024.120918. 39505226

[48] H. S. Yang , B. Inglis , T. M. Talavage , et al., “Coupling Between Cerebrovascular Oscillations and CSF Flow Fluctuations During Wakefulness: An fMRI Study,” Journal of Cerebral Blood Flow and Metabolism 42, no. 6 (2022): 1091–1103, 10.1177/0271678X221074639. 35037498 PMC9125495

[49] Y. Zhang , B. Peng , S. Chen , et al., “Reduced Coupling Between Global Signal and Cerebrospinal Fluid Inflow in Patients With Depressive Disorder: A Resting State Functional MRI Study,” Journal of Affective Disorders 354 (2024): 136–142, 10.1016/j.jad.2024.03.023. 38484877

[50] Z. Wang , Z. Song , C. Zhou , et al., “Reduced Coupling of Global Brain Function and Cerebrospinal Fluid Dynamics in Parkinson's Disease,” Journal of Cerebral Blood Flow and Metabolism 43, no. 8 (2023): 1328–1339, 10.1177/0271678X231164337. 36927139 PMC10369155

[51] V. Kiviniemi , X. Wang , V. Korhonen , et al., “Ultra‐Fast Magnetic Resonance Encephalography of Physiological Brain Activity—Glymphatic Pulsation Mechanisms?,” Journal of Cerebral Blood Flow and Metabolism 36, no. 6 (2016): 1033–1045, 10.1177/0271678X15622047. 26690495 PMC4908626

[52] A. M. Wright , Y. C. Wu , N. K. Chen , and Q. Wen , “Exploring Radial Asymmetry in MR Diffusion Tensor Imaging and Its Impact on the Interpretation of Glymphatic Mechanisms,” Journal of Magnetic Resonance Imaging 60, no. 4 (2024): 1432–1441, 10.1002/jmri.29203. 38156600 PMC11213825

