# Supplementary material for: The Relationship Between Glymphatic Function, White Matter Hyperintensity and Cognition: A Structural Equation Model MRI Study
Source: CNS Neurosci Ther. 2025 Jun 19;31(6):e70478. doi: 10.1111/cns.70478 (PMC12179404; doi:10.1111/cns.70478)
Supplement: Supplementary file 1 — Figure S1. Schematic of the study workflow showing methods for assessment of glymphatic circulation. Figure S2. Semiquantitative and fully quantitative evaluation of meningeal lymphatic vessel function via DCE‐MRI. Figure S3. Three sections of CSF signal extraction from the cerebellar substratum and box plot of the global BOLD–CSF coupling coefficient. Figure S4. Relationships of glymphatic circulation indicators with WMH and cognition. Table S1. Comparison of glymphatic circulation indicators among study groups. Table S2. Multivariate linear regression analysis of glymphatic inflow in the WMH group. Table S3. Multivariate linear regression analysis of glymphatic outflow in the WMH group. Table S4. Relationships between ChP/LatVent value and ChP permeability in each group. Text S1. MRI protocol used in our study. Text S2. MRI evaluation of enlarged perivascular space, lacunes, and microbleeds. [file CNS-31-e70478-s001.docx]

**Supplementary Materials**

**Supplementary Figure 1.** Schematic of the study workflow showing methods for assessment of glymphatic circulation

**Supplementary Figure 2.** Semiquantitative and fully quantitative evaluation of meningeal lymphatic vessel function via DCE-MRI

**Supplementary Figure 3.** Three sections of CSF signal extraction from the cerebellar substratum and box plot of the global BOLD–CSF coupling coefficient

**Supplementary Figure 4.** Relationships of glymphatic circulation indicators with WMH and cognition

**Supplementary Table 1.** Comparison of glymphatic circulation indicators among study groups

**Supplementary Table 2.** Multivariate linear regression analysis of glymphatic inflow in the WMH group

**Supplementary Table 3.** Multivariate linear regression analysis of glymphatic outflow in the WMH group

**Supplementary Table 4.** Relationships between ChP/LatVent value and ChP permeability in each group

**Supplementary Text 1.** MRI protocol used in our study

**Supplementary Text 2.** MRI evaluation of enlarged perivascular space, lacunes, and microbleeds


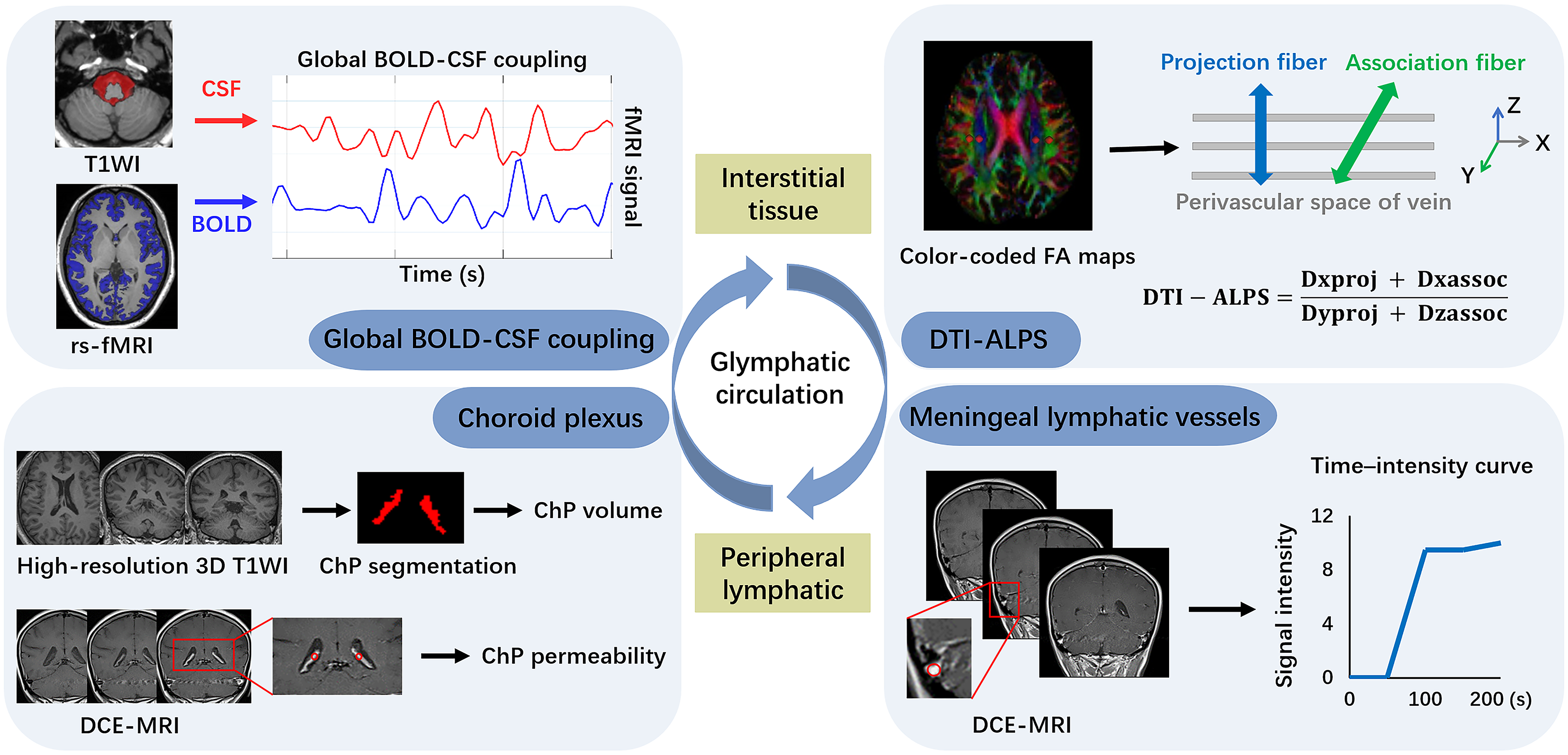


**Supplementary Figure 1. Schematic of the study workflow showing methods for assessment of glymphatic circulation**

Abbreviations: ChP = choroid plexus; DCE-MRI = dynamic contrast-enhanced magnetic resonance imaging; T1WI = T1-weighted imaging; rs-fMRI = resting-state functional magnetic resonance imaging; BOLD = blood-oxygen-level-dependent; CSF = cerebrospinal fluid; DTI-ALPS = diffusion tensor image analysis along the perivascular space; FA = fractional anisotropy; 3D = three-dimensional.


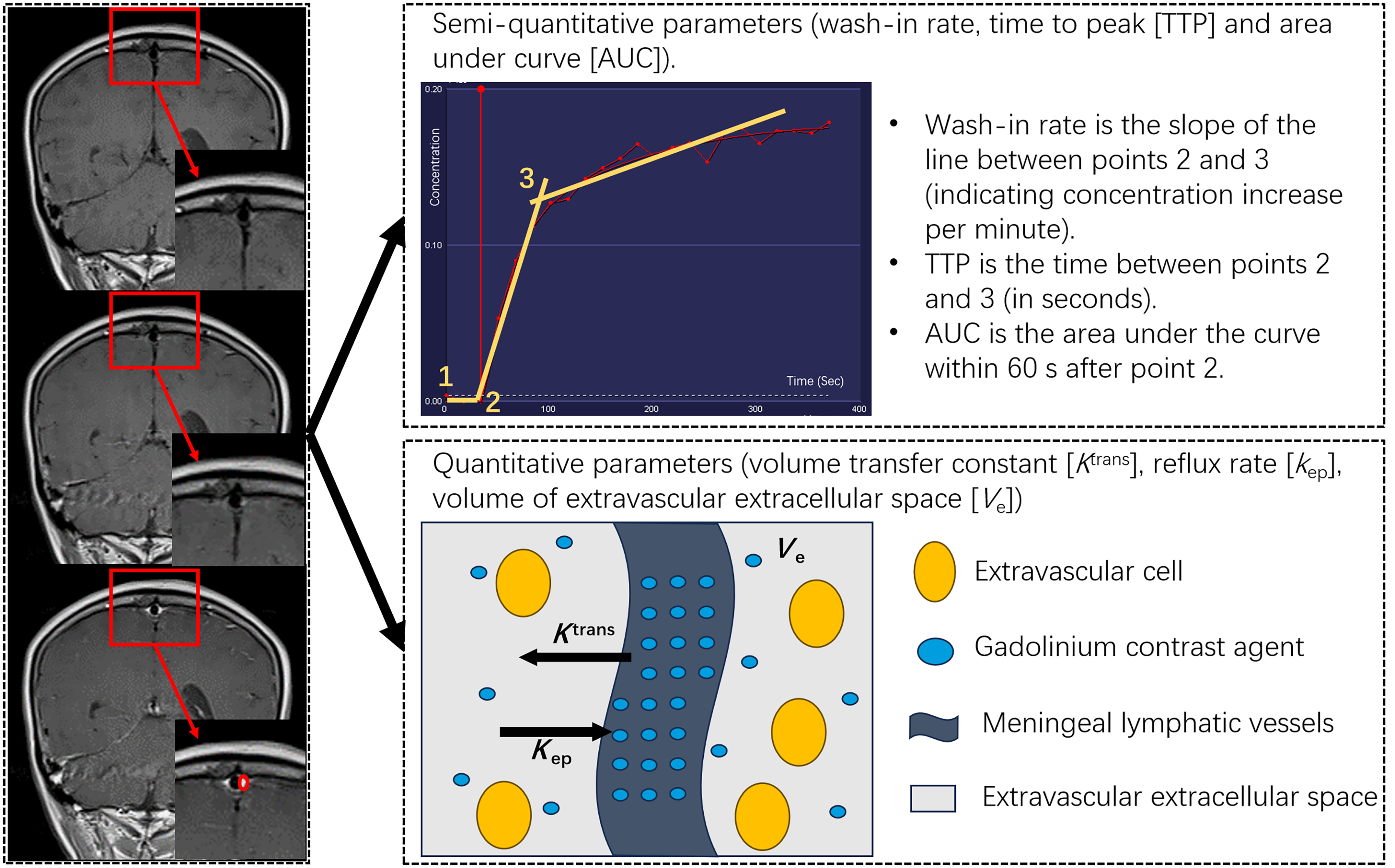


**Supplementary Figure 2. Semiquantitative and fully quantitative evaluation of meningeal lymphatic vessel function via DCE-MRI**


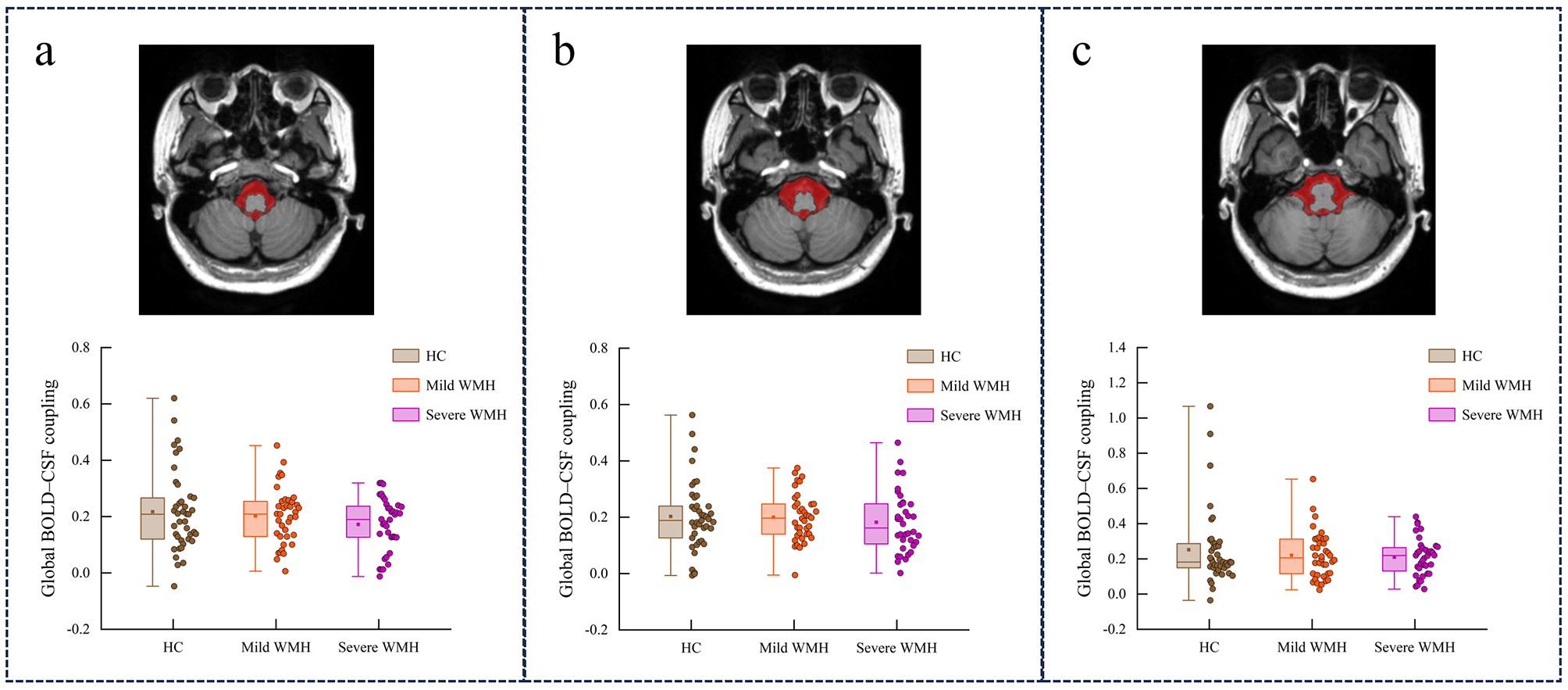


**Supplementary Figure 3. Three sections of CSF signal extraction from the cerebellar substratum and box plot of the global BOLD–CSF coupling coefficient**

No significant difference in coupling strength was observed among the three manually delineated regions of interest.


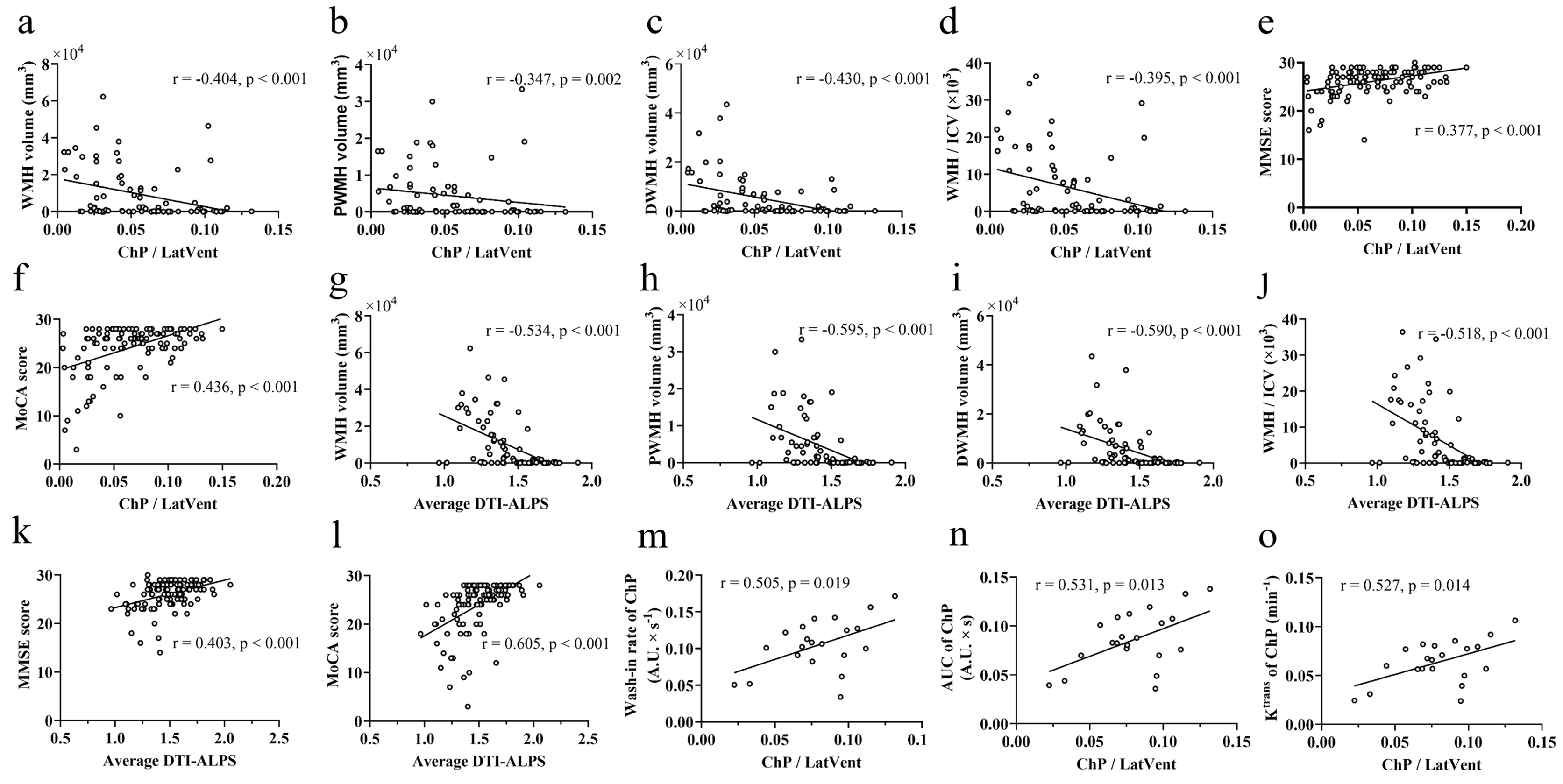


**Supplementary Figure 4. Relationships of glymphatic circulation indicators with WMH and cognition**

Abbreviations: WMH = white matter hyperintensity; DWMH = deep WMH; PWMH = periventricular WMH; ICV = intracranial volume; MMSE = Mini-Mental State Examination; MoCA = Montreal Cognitive Assessment; ChP = choroid plexus; LatVent = lateral ventricle; AUC = area under the curve; DTI-ALPS = diffusion tensor image analysis along the perivascular space.

**Supplementary Table 1. Comparison of glymphatic circulation indicators among study groups**

| **Variable** | **HC vs Mild WMH** | **HC vs Severe WMH** | **Mild WMH vs Severe WMH** |
| --- | --- | --- | --- |
| **ChP** |  |  |  |
| ChP volume | 0.054 | **0.008** | 0.414 |
| ChP/LatVent | 0.232 | **<0.001** | **<0.001** |
| ChP/ICV (×10^-3^) | 0.093 | **0.016** | 0.423 |
| Global BOLD–CSF coupling |  |  |  |
| Maximum strength | 0.570 | 0.092 | 0.266 |
| +2 s lag strength | 0.074 | 0.206 | 0.623 |
| **DTI-ALPS** |  |  |  |
| Left | 0.487 | **<0.001** | **<0.001** |
| Right | 0.707 | **<0.001** | **<0.001** |
| Bilateral average | 0.518 | **<0.001** | **<0.001** |
| **Dorsal MLVs** |  |  |  |
| wash–in rate | 0.391 | 0.077 | 0.350 |
| TTP | 0.113 | **0.009** | 0.354 |
| AUC | 0.058 | **0.010** | 0.560 |
| *K*^trans^ | 0.220 | 0.061 | 0.492 |
| *k*_ep_ | 0.203 | **0.011** | 0.186 |
| *V*_e_ | 0.890 | 0.104 | 0.137 |
| **Left basal MLVs** |  |  |  |
| wash–in rate | 0.306 | 0.451 | 0.083 |
| TTP | 0.588 | 0.726 | 0.383 |
| AUC | 0.577 | 0.247 | 0.092 |
| *K*^trans^ | 0.877 | 0.160 | 0.122 |
| *k*_ep_ | 0.713 | 0.030 | 0.080 |
| *V*_e_ | 0.836 | 0.942 | 0.785 |
| **Right basal MLVs** |  |  |  |
| wash–in rate | 0.158 | 0.112 | 0.830 |
| TTP | 0.504 | 0.076 | 0.283 |
| AUC | 0.145 | 0.077 | 0.724 |
| *K*^trans^ | 0.125 | 0.081 | 0.797 |
| *k*_ep_ | 0.230 | 0.229 | 0.972 |
| *V*_e_ | 0.258 | 0.398 | 0.799 |

*P* <0.05 after Bonferroni correction for multiple comparisons.

Abbreviations: HC = healthy control; WMH = white matter hyperintensity; ChP = choroid plexus; LatVent = lateral ventricle; ICV = intracranial volume; BOLD = blood oxygenation level dependent; CSF = cerebrospinal fluid; DTI-ALPS = diffusion tensor image analysis along the perivascular space; MLVs = meningeal lymphatic vessels; TTP = time to peak; AUC = area under the curve.

**Supplementary Table 2. Robust regression analysis of glymphatic inflow in the WMH group**

|  | Nonnormalized Coefficient | | Standardization Coefficient | *t* | *P* |
| --- | --- | --- | --- | --- | --- |
|  | B | SE | Beta |  |  |
| Age | 0.035 | 0.028 | 0.202 | 1.281 | 0.200 |
| Sex | -1.114 | 0.755 | -0.218 | -1.477 | 0.140 |
| Education | -0.198 | 0.106 | -0.244 | -1.878 | 0.060 |
| BMI | -0.037 | 0.117 | -0.033 | -0.318 | 0.750 |
| EPVS | 0.015 | 0.576 | 0.003 | 0.026 | 0.979 |
| Microbleeds | 0.622 | 0.681 | 0.102 | 0.914 | 0.361 |
| CP volume | 0.004 | 0.004 | 0.831 | 1.155 | 0.248 |
| CP/LatVent | -32.046 | 12.219 | -0.39 | -2.623 | **0.009** |
| ChP/ICV (×10^3^) | -3.702 | 5.647 | -0.473 | -0.656 | 0.512 |
| Global BOLD–CSF coupling Maximum strength | -4.264 | 2.861 | -0.162 | -1.491 | 0.136 |
| Global BOLD–CSF +2s lag strength | 1.326 | 2.208 | -0.094 | -0.601 | 0.548 |
| Global BOLD–CSF -6 s lag strength | -1.349 | 2.756 | -0.082 | -0.489 | 0.625 |
| d(globalBOLD)/dt coupling strength | 2.975 | 2.626 | 0.22 | 1.133 | 0.257 |

Dependent variable: total WMH volume/intracranial volume (following the logarithmic).

Abbreviations: EPVS = enlarged perivascular space; BMI = body mass index; ChP = choroid plexus; LatVent = lateral ventricle; ICV = intracranial volume.

**Supplementary Table 3. Robust regression analysis of glymphatic outflow in the WMH group**

|  | Nonnormalized Coefficient | | Standardization Coefficient | *t* | *P* |
| --- | --- | --- | --- | --- | --- |
|  | B | SE | Beta |  |  |
| Age | 0.025 | 0.024 | 0.145 | 1.066 | 0.286 |
| Sex | -0.915 | 0.515 | -0.179 | -1.775 | 0.076 |
| Education | -0.125 | 0.096 | -0.153 | -1.298 | 0.194 |
| BMI | 0.07 | 0.107 | 0.062 | 0.661 | 0.509 |
| EPVS | 0.625 | 0.474 | 0.122 | 1.318 | 0.187 |
| Microbleeds | 0.94 | 0.602 | 0.155 | 1.563 | 0.118 |
| Dorsal MLVs Wash-in | 1.57 | 37.436 | 0.018 | 0.042 | 0.967 |
| Dorsal MLVs TTP | -0.886 | 1.229 | -0.119 | -0.72 | 0.471 |
| Dorsal MLVs AUC | -19.946 | 36.767 | -0.184 | -0.542 | 0.587 |
| Dorsal MLVs *K*^trans^ | 2.843 | 48.73 | 0.018 | 0.058 | 0.953 |
| Dorsal MLVs *k*_ep_ | 1.384 | 2.301 | 0.08 | 0.602 | 0.547 |
| Dorsal MLVs *V*_e_ | 0.636 | 3.143 | 0.028 | 0.202 | 0.84 |
| Average DTI-ALPS | -6.628 | 1.55 | -0.531 | -4.277 | **0.000** |

Dependent variable: total WMH volume/intracranial volume (following the logarithmic).

Abbreviations: EPVS = enlarged perivascular space; BMI = body mass index; MLVs = meningeal lymphatic vessels; TTP = time to peak; AUC = area under curve; DTI-ALPS = diffusion tensor image analysis along the perivascular space.

**Supplementary Table 4. Relationships between ChP/LatVent value and ChP permeability in each group**

| **Variable** | **Total (n=62)** | **HC (n=23)** | **Mild WMH (n=21)** | **Severe WMH (n=18)** |
| --- | --- | --- | --- | --- |
| ChP average_Wash-in | 0.230 (0.073) | -0.293 (0.174) | **0.505 (0.019)** | 0.172 (0.495) |
| ChP average_TTP | 0.149 (0.247) | 0.271 (0.212) | 0.193 (0.401) | -0.057 (0.824) |
| ChP average_AUC | 0.241 (0.059) | -0.247 (0.257) | **0.531 (0.013)** | 0.214 (0.395) |
| ChP average_*K*^trans^ | **0.257 (0.044)** | -0.230 (0.290) | **0.527 (0.014)** | 0.167 (0.509) |
| ChP average_ *k*_ep_ | 0.094 (0.466) | -0.218 (0.317) | 0.350 (0.120) | -0.140 (0.579) |
| ChP average_*V*_e_ | 0.172 (0.181) | -0.158 (0.471) | 0.116 (0.616) | 0.194 (0.442) |

Note: Statistical results are reported as correlation coefficients (*P* values).

Abbreviations: ChP = choroid plexus; LatVent = lateral ventricle; TTP = time to peak; AUC = area under the curve.

**Supplementary Text 1. MRI protocol used in our study**

High-resolution three-dimensional T1-weighted imaging: repetition time (TR)/echo time (TE) = 1900/2.26 ms, NEX = 1, acquisition matrix = 240 × 256, field of view (FOV) = 215 × 230 mm, number of slices = 176, and slice thickness = 1.0 mm. T2-weighted imaging: TR/TE = 4000 ms/113 ms, acquisition matrix = 320 × 298, FOV = 220 × 220 mm, and slice thickness = 5 mm. T2-FLAIR: TR/TE = 7000 ms/79 ms, acquisition matrix = 256 × 256, FOV = 200 × 200 mm, and slice thickness = 5 mm. Resting-state functional MRI (rs-fMRI) was performed using a standard T2*-weighted gradient echo sequence with the following parameters: TR/TE = 2000/30 ms, flip angle = 90°, FOV = 200 × 200 mm, matrix = 64 × 64, slice thickness = 4 mm, interslice gap = 1.2 mm, and number of time points = 190. During rs-fMRI scanning, participants were instructed to keep their eyes closed, avoid specific thoughts, and remain awake. DTI: number of slices = 49, slice thickness = 2.5 mm, TR/TE = 7200 ms/104 ms, NEX = 2, matrix = 128 × 128, FOV = 230 × 230 mm; 32 acquisitions using diffusion encoding (b = 1000 s/mm²) and without diffusion encoding (b = 0 s/mm²), total acquisition time = 8 min 11 s. The DCE-MRI protocol utilized a standard two-dimensional T1 black-blood sequence with coronal two-dimensional acquisition. Number of series = 23; each series lasted 16.78 s and included three contiguous slices with thickness = 3 mm, TR/TE = 700 ms/11 ms, FOV = 200 × 200 mm, matrix = 154 × 192, voxel size = 0.9 × 0.9 × 3.0 mm, and total acquisition time = 6 min 26 s.

**Supplementary Text 2. MRI evaluation of enlarged perivascular space, lacunes, and microbleeds**

In this study, the MRI-visible perivascular space (PVS) was defined as an enlarged PVS (EPVS) if it met the following criteria: 1) signal intensity similar to CSF across all sequences; 2) alignment with the direction of the penetrating blood vessel, appearing either linear (parallel to the vessel) or round/ovoid (perpendicular to the vessel); 3) no enhancement effect; and 4) a maximum diameter of < 3 mm. T2-weighted axial MR sequences were primarily used to determine EPVS number, in accordance with a validated method. Lacunes were defined as focal lesions measuring 3–15 mm with signal characteristics identical to CSF across all sequences. Microbleeds were defined as 2–10 mm round or ovoid hypointense lesions on susceptibility-weighted imaging. All MRI markers were identified according to established standards for reporting vascular changes on neuroimaging. The presence and number of EPVS, as well as the presence of lacunes and microbleeds, were recorded by authors Lin Wu and Kaixiao Chen.
